# Contamination of street food with multidrug-resistant *Salmonella*, in Ouagadougou, Burkina Faso

**DOI:** 10.1371/journal.pone.0253312

**Published:** 2021-06-17

**Authors:** Marguerite E. M. Nikiema, Maria Pardos de la Gandara, Kiswensida A. M. Compaore, Absétou Ky Ba, Karna D. Soro, Philippe A. Nikiema, Nicolas Barro, Lassana Sangare, François-Xavier Weill

**Affiliations:** 1 Laboratoire d’Epidémiologie et de Surveillance des Bactéries et Virus transmissibles par les Aliments, Ecole Doctorale Sciences et Technologie (EDST), Université Joseph Ki-Zerbo, Ouagadougou, Burkina Faso; 2 Centre National de Référence des *Escherichia coli*, *Shigella* et *Salmonella*, Unité des Bactéries Pathogènes Entériques, Institut Pasteur, Paris, France; 3 Laboratoire National de Santé Publique, Ouagadougou, Burkina Faso; 4 Unité de Formation et de Recherche en Sciences de la Santé (UFR/SDS)/Ecole Doctorale Sciences et Santé (EDSS), Université Joseph Ki-Zerbo, Ouagadougou, Burkina Faso; 5 Laboratoire de Bactériologie-Virologie, Centre Hospitalier Universitaire Yalgado Ouédraogo, Ouagadougou, Burkina Faso; St Petersburg Pasteur Institute, RUSSIAN FEDERATION

## Abstract

**Background:**

Gastrointestinal infections are a global public health problem. In Burkina Faso, West Africa, exposure to *Salmonella* through the consumption of unhygienic street food represents a major risk of infection requiring detailed evaluation.

**Methods:**

Between June 2017 and July 2018, we sampled 201 street food stalls, in 11 geographic sectors of Ouagadougou, Burkina Faso. We checked for *Salmonella* contamination in 201 sandwiches (one per seller), according to the ISO 6579:2002 standard. All *Salmonella* isolates were characterized by serotyping and antimicrobial susceptibility testing, and whole-genome sequencing was performed on a subset of isolates, to investigate their phylogenetic relationships and antimicrobial resistance determinants.

**Results:**

The prevalence of *Salmonella enterica* was 17.9% (36/201) and the *Salmonella* isolates belonged to 16 different serotypes, the most frequent being Kentucky, Derby and Tennessee, with five isolates each. Six *Salmonella* isolates from serotypes Brancaster and Kentucky were multidrug-resistant (MDR). Whole-genome sequencing revealed that four of these MDR isolates belonged to the emergent *S*. *enterica* serotype Kentucky clone ST198-X1 and to an invasive lineage of *S*. *enterica* serotype Enteritidis (West African clade).

**Conclusion:**

This study reveals a high prevalence of *Salmonella* spp. in sandwiches sold in Ouagadougou. The presence of MDR *Salmonella* in food on sale detected in this study is also matter of concern.

## Introduction

Salmonellosis is one of the main foodborne diseases worldwide. The main reservoirs of *Salmonella* spp. implicated in food poisoning are contaminated foods, such as meat, eggs, milk, seafood, fruits and vegetables [1–4]. Population growth and continual rapid urbanisation have imposed new dietary habits on the population of Burkina Faso, with the emergence of so-called “street food” habits, in particular [5,6]. Eighty per cent of the urban population in Burkina Faso regularly consumes street food, including diverse types of sandwiches, including vegetables, beef, chicken and egg products [5]. Street food has developed principally to improve the nutritional value of traditional food and to meet consumer needs (culturally and socioeconomically). However, it has also raised major issues, such as the need to respect sanitary requirements to prevent collective food poisoning incidents [7]. Foodborne diseases can cause significant morbidity and mortality in both humans and livestock, and considerable economic losses [8,9]. The intensive use of antibiotics in humans and animals has favoured the emergence of resistance in various bacterial pathogens, including *Salmonella* spp. [10]. Antibiotics have been widely used in animal production, not only to prevent and treat bacterial infections, but also, notably, as a growth promotor [11,12]. These industrial animal production practices have turned food animals into a major reservoir of *Salmonella* spp. resistant to antibiotics [13]. Some multidrug-resistant (MDR) *Salmonella* populations, such as the “ST198-X1” clone of serotype Kentucky [14] or the invasive clones of serotypes Enteritidis [15] and Typhimurium [16] have spread throughout Africa. Previous studies in Burkina Faso have shown that *Salmonella enterica* strains isolated from raw beef meat, chicken meat and faeces, and from eggs were resistant to commonly used antibiotics [2,3,13], but the genetic relationships between these strains and known MDR populations circulating at a larger scale were not assessed.

Bacterial whole-genome sequencing (WGS) is rapidly gaining importance for epidemiological investigations of infections and outbreaks. New genomic approaches have been developed, allowing a more thorough study of *Salmonella* populations. One such approach is core genome multilocus sequence typing (cgMLST), in which *Salmonella* strains are typed on the basis of the sequences of 3 002 genes present in the core genome [17]. Hierarchical clustering on the basis of cgMLST data results in genomic clustering into 13 levels: from HC0, in which strains are at a distance of 0 to 2 alleles, to HC2850, predictive of *Salmonella* subspecies [18]. EnteroBase (http://enterobase.warwick.ac.uk/) is a software environment providing these and other tools for genomic analyses, together with a vast collection (> 275 000) of genomic assemblies for *Salmonella* spp., contributed by laboratories from all over the world [19]. These methods can be used to determine whether bacterial isolates belong to previously described clusters, and to estimate epidemiological relationships between isolates.

In this study, we assessed the contamination of sandwiches with *Salmonella* spp., to determine the role of this sort of street food as a vehicle of *Salmonella* transmission in Ouagadougou. The characteristics of the *Salmonella* strains isolated, including genomic diversity and antimicrobial susceptibility in particular, were also determined.

## Materials and methods

### Sampling

Between June 2017 and July 2018, we sampled 201 street food stalls, in 11 geographic sectors of six different districts of Ouagadougou, the capital of Burkina Faso (S1 Fig). The geographic sectors selected for this study were those with the highest population densities; they were also the sectors with the largest numbers of schools, markets and hospitals. We collected 201 sandwiches (one sandwich per seller) (Table 1), at random, from the various sites. Each individual sample was bought, packed in a sterile bag and placed in a container at +4°C for transfer to the laboratory. Each sample was labelled with the selling area and type of sandwich.

**Table 1:** Composition of the sandwiches analysed.

Type of sandwich (*n*)	Description
Beef kilichi (20)	• Bread • Beef kilichi: thin strips of beef. • Seasoning: these strips are coated with a peanut paste and various condiments and vegetables, including onion, chilli pepper and other spices and roasted on charcoal.
Beef kebab (102)	• Bread • Beef kebab: small pieces of beef threaded onto wooden skewers and braised on charcoal • Seasoning: chilli pepper, mayonnaise, raw vegetables (tomatoes, onions, cucumbers, parsley), ketchup, mustard
Minced beef (18)	• Bread • Minced beef cooked with various ingredients (parsley, garlic, chilli pepper, onions etc.), then crumbled
Fish (27)	• Bread • Fish cooked with various ingredients (parsley, garlic, chilli pepper, onion etc.), then crumbled
Omelette (18)	• Bread • Fried eggs • - Seasoning: onions and tomatoes
Avocado (12)	• Bread • Avocado • Seasoning: raw vegetables, oil, and broth powder
Sausage (4)	• Bread • Sausage • Seasoning: mayonnaise, ketchup

*n*: Number of sandwiches of each type.

### *Salmonella* spp. identification

The *Salmonella* strains in the samples were isolated and identified according to the methods of the ISO 6579:2002 standard (updated in 2007) “Horizontal method for detection of *Salmonella* spp.” This detection process has four stages, as previously described [20,21]. *S*. *enterica* serotype Typhimurium strain ATCC 14028 and *S*. *enterica* serotype Enteritidis strain ATCC 13076 were used as positive controls. Suspected colonies (one per sample) were purified on nutrient agar and analysed with the API 20E (BioMérieux, Marcy l’Etoile, France) panel, for biochemical confirmation. Isolates of *Salmonella* spp. were stored in brain heart broth (BioMérieux) supplemented with 30% glycerol, in cryotubes at -80°C.

### Serotyping

Serotyping was performed at the French National Reference Centre for *Escherichia coli*, *Shigella* and *Salmonella* (CNR-ESS) at the Institut Pasteur in Paris, France. *Salmonella* spp. isolates were serotyped according to the White-Kauffmann-Le Minor scheme [22]. Sera from Bio-Rad (Marnes-la-Coquette, France), Statens Serum Institut (Copenhagen, Denmark) and in-house sera from the CNR-ESS were used for this purpose.

### Antimicrobial susceptibility testing

Antimicrobial susceptibility was determined by the disk diffusion method, on Mueller-Hinton (MH) agar, in accordance with the guidelines of the Antibiogram Committee of the French Society for Microbiology [23]. The following 22 antimicrobial drugs (Bio-Rad) were tested: ampicillin (AMP, 10 μg), amoxicillin plus clavulanic acid (AMC, 30 μg), cefoxitin (FOX, 30 μg), cefotaxime (COX, 5 μg), ceftazidime (CZD, 10 μg), cefepime (FEP, 30 μg), streptomycin (SMN, 10 μg), spectinomycin (SPT, 100 μg), gentamicin (GEN, 10 μg), amikacin (AKN, 30 μg), tigecycline (TGC, 15 μg), kanamycin (KAN, 30 μg), sulfonamides (SSS, 200 μg), trimethoprim (TMP, 5 μg), trimethoprim-sulfamethoxazole (SXT, 25 μg), chloramphenicol (CHL, 30 μg), tetracycline (TET, 30 μg), nalidixic acid (NAL, 30 μg), ciprofloxacin (CIP, 5 μg), pefloxacin (PEF, 5 μg), meropenem (MEM, 10 μg), and azithromycin (AZM, 15 μg). The minimum inhibitory concentrations (MICs) of NAL and CIP were determined by E-tests (BioMérieux) on NAL-resistant isolates identified by the disk diffusion method. The recommended reference strain *Escherichia coli* ATCC 25922 was used as a control for antibiotic susceptibility testing. This technique was performed at the CNR-ESS.

### Whole-genome sequencing

Twelve *Salmonella* isolates from the most frequent serotypes recovered in this study—Agona (*n* = 1), Derby (*n* = 5), Enteritidis (*n* = 1), and Kentucky (*n* = 5)—were selected for whole-genome sequencing (WGS).

Total DNA was extracted with the MagNAPure 96 system robot (Roche), from overnight cultures in tryptic soy broth (TSB) at 37°C. WGS was performed on the genomic platform of the Institut Pasteur, in Paris, France (“Plateforme de microbiologie mutualisée”, P2M). The libraries were prepared with the Nextera XT kit (Illumina) and sequencing was performed with the NextSeq 500 system (Illumina), generating 150 bp paired-end reads. The short reads were assembled *de novo* with SPAdes version 3.6.0.23 [24].

Short-read sequence data were submitted to EnteroBase (http://enterobase.warwick.ac.uk/) [17] and to the European Nucleotide Archive (ENA) (http://www.ebi.ac.uk/ena), under study accession number PRJEB44192; the genome accession numbers are provided in S1 Table.

Serotype prediction, MLST [25], and core genome MLST (cgMLST) were performed with various tools integrated into EnteroBase. Phylogenetic information was obtained by applying the EnteroBase HierCC scheme to cgMLST [18].

The presence and type of antimicrobial resistance genes (ARGs) or ARG-containing structures were determined with ResFinder version 4.1 (https://cge.cbs.dtu.dk/services/ResFinder/) [26] and PlasmidFinder version 1.3 (https://cge.cbs.dtu.dk/services/PlasmidFinder/) [27] on SPAdes assemblies.

A comparison between the serotype Enteritidis isolate MARG-18AL-BROO identified in our study and the invasive MDR African populations of *S*. *enterica* serotype Enteritidis recently described by Feasey et al. [15] was performed with EnteroBase. This database performs regular scans of the GenBank Sequence Read Archive (SRA), uploads the new short-read sequences for several pathogens, and assembles the short-reads into annotated draft genomes. The “Custom View” utility can be used to upload additional metadata for genomes already present on the site. Five-hundred and three (503) of the 677 genomes analysed by Feasey et al. [15] are available from EnteroBase. A Custom View was created, including the hierBAPS clade/cluster data described by Feasey et al. [15], to compare the hierBAPS clustering with the Enterobase HierCC clustering (S2 Table). A minimum spanning (MS) tree (MStree V2 or GrapeTree) based on the EnteroBase “cgMLST V2 + HierCC V1” scheme was produced to estimate the allelic distances between MARG-18AL-BROO and the *S*. *enterica* serotype Enteritidis genomes from Feasey et al. [15].

## Results

### *Salmonella* contamination

**Fig 1:**
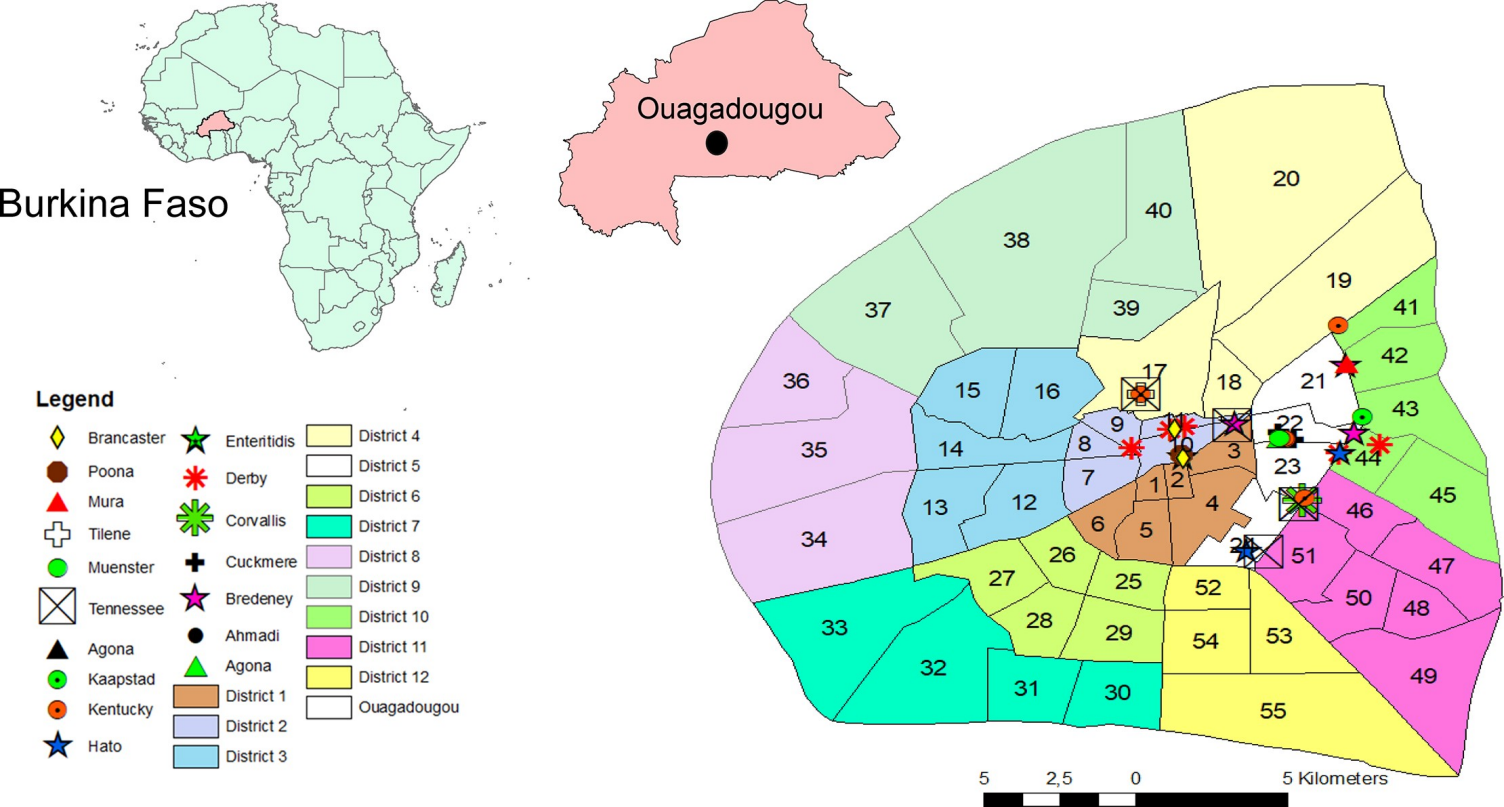
Geographic location of the various *Salmonella* serotypes identified in our study.

Thirty-six (36) of the 201 (17.9%) sandwich samples analysed were contaminated with *Salmonella* spp. In total, 18 (50%) of these contaminated sandwiches were made with beef kebabs, six (16.7%) with fish, five (13.9%) with beef kilichi, three (8.3%) with omelette, three (8.3%) with minced beef meat, and one (2.8%) with avocado. No *Salmonella* spp. strains were detected in sausage sandwiches. Antigenic characterisation of the 36 *Salmonella* isolates led to the identification of 16 distinct serotypes (Fig 1, Tables 2 and S1). Two isolates were “rough” (they could not be serotyped due to auto-agglutination). Kentucky, Derby and Tennessee were the most frequent serotypes, with five isolates each (Table 2).

**Table 2:** Serotypes of *Salmonella enterica* isolated from sandwich samples and their antibiotic resistance profiles.

	No. of isolates per type of sandwich							
Serotype	Avocado (*n* = 12)	Fish (*n* = 27)	Kilichi (*n* = 20)	Minced beef (*n* = 18)	Omelette (*n* = 18)	Kebab (*n* = 102)	Total (*n* = 201)	AMR profile*(*n*)
Agona					1		1	
Ahmadi						1	1	
Brancaster			1			1	2	SMN-KAN-SSS-TMP-SXT-TET (2)
Bredeney						3	3	
Corvallis						1	1	
Cuckmere					1	2	3	
Derby		1	3			1	5	
Enteritidis						1	1	AMP-SMN-SSS-CHL (1)
Hato		1				1	2	
Kaapstad					1		1	
Kentucky		1				4	5	AMP-SMN-SPT-GEN-SSS-TET-NAL-CIP (3)
Muenster						1	1	
Mura						1	1	
Poona						1	1	
Tennessee		3		2			5	
Tilene			1				1	
“Rough”	1			1			2	
**Total isolates n (%)**	**1 (8.3%)**	**6 (22.3%)**	**5 (25.0%)**	**3 (16.6%)**	**3 (16.6%)**	**18 (17.6%)**	**36 (17.9%)**	

*abbreviations: AMR, antimicrobial resistance; AMP, ampicillin; SMN, streptomycin; SPT, spectinomycin; GEN, gentamicin; SSS, sulfonamides; TMP, trimethoprim; SXT, trimethoprim-sulfamethoxazole; CHL, chloramphenicol; TET, tetracycline; NAL, nalidixic acid; CIP, ciprofloxacin.

### Molecular sub-typing

Twelve *Salmonella* isolates were selected for whole-genome sequencing (WGS): Agona (*n* = 1), Derby (*n* = 5), Enteritidis (*n* = 1), and Kentucky (*n* = 5). The results are provided in S1 Table.

Five new MLST profiles were obtained for isolates of serotypes Agona (ST7876), Derby (ST7881, ST7882, ST7889), and Kentucky (ST7879). The serotype Enteritidis isolate belonged to ST11, and three isolates of serotype Kentucky were ST198.

The serotype Agona isolate belonged to the HC2000_12 superlineage according to the EnteroBase HierCC scheme [18]. No other serotype Agona sequences available from EnteroBase were closer than HC900 for this isolate.

The five serotype Derby isolates belonged to three different superlineages: HC2000_26666 (more than 20 genomes in EnteroBase), HC2000_181877 (common to one isolate obtained in 2019 in France from EnteroBase), and a newly identified HC2000_227639 superlineage.

**Fig 2:**
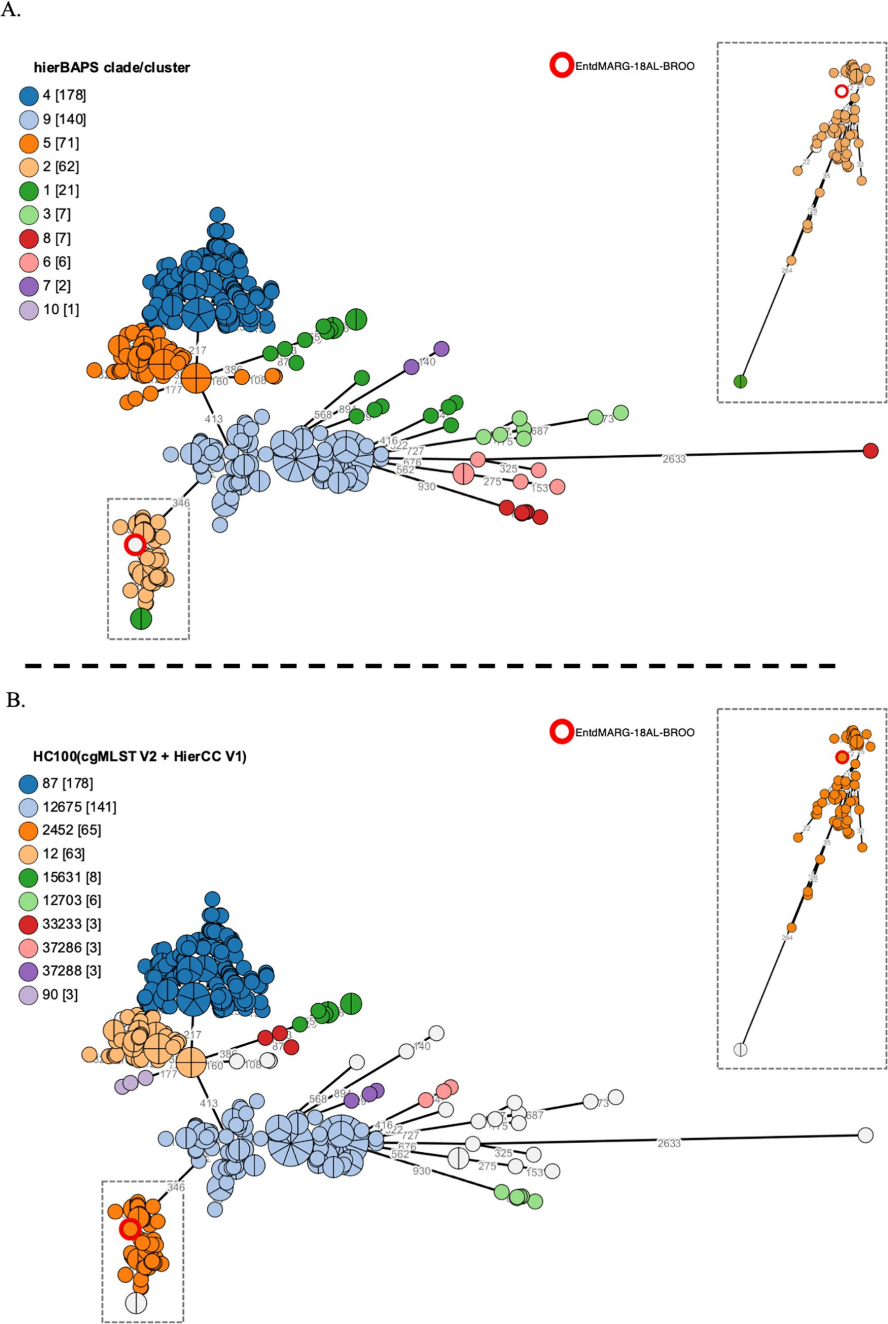
Genomic clustering of the *S*. *enterica* serotype Enteritidis genome identified in this study. Minimal spanning tree generated by the EnteroBase “MSTree V2” algorithm and including all503 *S*. *enterica* serotype Enteritidis genomes from Feasey et al. [15] available from EnteroBase, and the *S*. *enterica* serotype Enteritidis genome from Burkina Faso (MARG-18AL-BROO, circled in red). Branch numbers correspond to allelic distances. The inset is a magnification of the branch containing isolate MARG-18AL-BROO. 3a. Grape colours correspond to the hierBAPS method performed by Feasey et al. [15]. 3b. Grape colours correspond to the HC100 assigned by the HierCC method performed in EnteroBase.

The serotype Enteritidis isolate (MARG-18AL-BROO) belonged to the HC2000_12 superlineage and to HC100_2452. We compared isolate MARG-18AL-BROO and the invasive MDR African populations of *S*. *enterica* serotype Enteritidis recently described by Feasey et al. [15]. The various *S*. *enterica* serotype Enteritidis clades in this previous study were defined by hierarchical Bayesian analysis of population structure (hierBAPS). All hierBAPS cluster 2 (also labelled as the West African clade) genomes from the study of Feasey et al. [15] available from EnteroBase (*n* = 62) belonged to HC100_2452. Our single isolate of serotype Enteritidis, recovered from a beef kebab sandwich, therefore belonged to the invasive MDR West African clade (Fig 2).

The five serotype Kentucky isolates clustered into two different superlineages: HC2000_528 and HC2000_7570 separated by a distance of more than 2 700 alleles (S1 Table). The three MDR serotype Kentucky isolates (see below) were ST198, clustered into the HC2000_528 superlineage, and shared a common HC20_528 (a very extended cluster, with more than 1 500 representative genomes in EnteroBase), with a maximum distance of 24 alleles between them. The two susceptible serotype Kentucky isolates clustered into the HC2000_7570 superlineage and shared a common HC20_227634, with a distance of eight alleles between them.

### Antimicrobial resistance

The 36 *Salmonella* isolates were tested against 22 antimicrobial drugs. Six (16.7%) of the 36 *Salmonella* isolates were resistant to four or more antibiotics; the remaining 30 *Salmonella* isolates (83.3%) were susceptible to all antibiotics tested (Table 2).

The six MDR isolates belonged to serotypes Kentucky (*n* = 3), Brancaster (*n* = 2), and Enteritidis (*n* = 1); no resistance was identified in the other isolates. The six MDR isolates were resistant to streptomycin (*strA* and *strB* for the four MDR isolates sequenced, belonging to serotypes Kentucky and Enteritidis), and sulfonamides (*sul1* for the serotype Kentucky isolates; *sul2* for the serotype Enteritidis isolate). Four MDR isolates (three serotype Kentucky and one serotype Enteritidis) were resistant to ampicillin (*bla*_TEM-1B_). The MDR serotype Enteritidis isolate was also resistant to chloramphenicol (*catA1*), whereas the two serotype Brancaster isolates and three serotype Kentucky isolates were also resistant to tetracycline (*tetA* for serotype Kentucky isolates). Only the serotype Kentucky isolates were resistant to fluoroquinolones, due to the accumulation of mutations in the topoisomerase genes (*gyrA* S83F and D87Y; *parC* S80I); they were also resistant to gentamicin (*aad7*). In addition, the *fosA7* gene, which confers resistance to fosfomycin (drug not tested by the disk diffusion method in our study), was identified in one serotype Agona isolate and two serotype Derby isolates. The presence of the *qacE*Δ1 gene, encoding resistance to quaternary ammonium compounds, in addition to *sul1*, suggested the presence of class 1 integrons in the three MDR Kentucky isolates (see S1 Table).

The MDR serotype Enteritidis isolate contained an IncI1 plasmid. No plasmids were found in the three MDR serotype Kentucky isolates. The two MDR serotype Brancaster isolates were not sequenced, and the presence of plasmids in these isolates was not, therefore, investigated.

## Discussion

We found that 17.9% of the sandwiches sampled at street food establishments in Ouagadougou, Burkina Faso were contaminated with *Salmonella* spp. In Egypt, Hassanin et al. [28] isolated *Salmonella* spp. from 31% of “shawarma-type” (kebab) sandwiches analysed, whereas Abd-El-Malek [29] isolated *Salmonella* from 7% of kibda-type sandwiches sampled. On the contrary, Djibrine et al. [30] isolated no *Salmonella* spp. from minced beef sandwich samples in Chad. These variations in contamination can be explained by regional variations in animal and environmental reservoirs of *Salmonella* spp. and sandwich preparation conditions.

The 36 sandwich samples contaminated with *Salmonella* in our study included 18 beef kebab sandwiches (50%) and five beef kilichi sandwiches (13.9%). A list of the types of sandwiches sold showed that beef kebab sandwiches were the most frequently prepared, available and consumed in Ouagadougou. The high frequency of kebab and kilichi samples (ranked first and third, respectively) and the different ingredients used to prepare these sandwiches may partly explain these results. In particular, these sandwiches were prepared with meat products (beef) widely recognised as reservoirs of *Salmonella*. In Burkina Faso, *Salmonella* has been isolated from 27% of raw beef meat and 18% of raw beef intestine samples [1]. Several studies have also reported poor hygiene conditions for the sale of raw beef, chicken and other ingredients in market places [1]. Raw beef meat may already be contaminated with *Salmonella*, which is then not completely destroyed during the grilling process, because the temperature may not be high enough or the cooking time too short. Another study conducted in Burkina Faso showed that 31% of the raw ingredients used to prepare sandwiches were contaminated with *Salmonella* from the water used to wash the lettuce [31]. The role of poultry meat as a vector of *Salmonella* was not assessed in our study, due to the lack of sandwiches containing poultry meat in our sample. This may reflect the low popularity of such sandwiches, which are rarely bought due to their high cost (three times more expensive than a beef kebab sandwich). However, poultry meat is manipulated by street food sellers, as this meat, grilled, braised or cooked in a soup is often consumed in the evening. Furthermore, the design of our study (random selection of one sandwich per seller) made it difficult to determine exactly how the *Salmonella* was introduced into the sandwiches. It could have come from any of the sandwich ingredients, cross-contamination or faecal contamination of the food handler’s hands.

The 36 *Salmonella* isolates identified here belonged to 16 different serotypes of *S*. *enterica* subspecies *enterica*. Serotypes Kentucky, Derby, and Tennessee were the most frequent. As we studied only one *Salmonella* colony per sample, we were not able to identify cases of sandwich contamination with several different serotypes in this study. Twelve of these 16 serotypes have already been detected in food animals or edible vegetables in Burkina Faso, between 2008 and 2018: Agona (cattle, poultry, fish), Brancaster (cattle, poultry), Bredeney (cattle, poultry, fish, lettuce), Derby (poultry, fish, sheep), Enteritidis (poultry), Hato (cattle, poultry), Kaapstad (poultry, pigs), Kentucky (poultry, fish), Muenster (cattle, poultry, pigs, fish, hedgehogs), Poona (cattle, poultry, fish), Tennessee (cattle), and Tilene (cattle, poultry, fish) [2–4,31–33]. Eight of these 16 serotypes were also reported in humans in this country (Bredeney, Derby, Enteritidis, Kentucky, Muenster, Poona, Tilene, and Tennessee) [34–36]. Most *Salmonella* serotypes are polyphyletic (i.e., formed by at least two unrelated lineages that convergently acquired the genetic basis for a similar O and H antigenic formula) [25]. Information restricted to the distribution of serotypes by source is not, therefore, sufficient for traceback investigations. Microbial genomics methods provide an accurate means of determining the phylogenetic relationships between isolates from the same *Salmonella* serotype.

*S*. *enterica* serotype Kentucky has been closely linked to poultry since 1937 [37], following its first isolation from chicken in the United States [38]. The three MDR S. *enterica* serotype Kentucky isolates identified in our study belonged to the “ST198-X1” clone [14]. This MDR clone, which is resistant to ciprofloxacin in particular, emerged in Egypt and spread throughout Africa and the Middle East from 2002, and has now become a serious public health problem worldwide [37,39]. Poultry has been identified as a major vehicle for infection by this MDR clone [37,39]. However, it has also been found in seafood, spices, pets and wild animals [39]. The two susceptible serotype Kentucky isolates sequenced here did not belong to this clone, but to the new ST7879 clone (S1 Table), suggesting a local or regional origin.

The main reservoirs of *S*. *enterica* serotype Derby are pigs and poultry worldwide [40]. As shown in S1 Table, the five serotype Derby isolates recovered from sandwiches in Ouagadougou clustered into three new MLST types: ST7881 (*n* = 2), ST7882 (*n* = 1), and ST7889 (*n* = 2), also suggesting a local or regional origin. These STs were unrelated to previously described serotype Derby isolates from pigs in France (ST39, ST40, and ST682). ST7881 and ST7889 are also five and six alleles distant, respectively, from poultry-associated ST71. ST7881 and ST7889 are also three alleles distant from ST683 (ST7881) and ST813 (ST7889), which have been reported for serotype Derby isolates from eggs in China [41] and from humans in France [40].

Two of the *S*. *enterica* serotype Derby ST7881 isolates from our study, and the only serotype Agona isolate identified, carried the *fosA7* gene conferring resistance to fosfomycin. This *fosA7* gene has been detected in *S*. *enterica* isolates from various sources across the globe, including serotype Derby and Heidelberg isolates from refuge dogs in Texas [42], serotype Agona isolated from chicken meat in Singapore [43], and serotype Derby ST39 isolated from humans and pigs in France [40]. To the best of our knowledge, this is the first time that *fosA7* has been described in *Salmonella* isolates from Africa.

Five serotype Tennessee isolates were identified in our study. This serotype was responsible for major outbreaks of salmonellosis in the US in 2007 and 2011, due to contaminated peanut butter [44,45]. Peanut paste is frequently used as a condiment in most of the sandwiches served in Ouagadougou (Table 1). However, we did not sequence the serotype Tennessee isolates recovered during our study to assess their phylogenetic relationships with the isolates from the peanut butter outbreaks in the US.

Feasey et al. [15] used WGS to study 675 isolates of *S*. *enterica* serotype Enteritidis from 45 countries, and revealed the existence of a global epidemic clade and two invasive clades restricted to different regions of Africa. These African invasive isolates showed a pattern of genomic degradation characteristic of adaptation to human hosts. Our single strain of serotype Enteritidis (MARG-18AL-BROO), belonged to the invasive MDR West African clade. Feasey et al. [15] also described a MDR plasmid, pSEN-BT, in the West African clade of *S*. *enterica* serotype Enteritidis. Strains from this clade had various antimicrobial drug resistance profiles, but their plasmids characteristically carried the chloramphenicol resistance gene, *catA1* –as observed for serotype Enteritidis in our study–whereas strains from Central and East Africa carried *catA2* [15].

In conclusion, a large proportion of sandwich samples (17.9%) in Ouagadougou displayed contamination with *Salmonella* spp., constituting a major potential source of consumer infection. Public health challenges due to foodborne contamination with *Salmonella* are aggravated by antibiotic resistance. Indeed, six of the 36 *Salmonella* isolates recovered from sandwiches here (16.67%) were MDR, including four isolates from emerging or invasive bacterial populations. The “One health” approach is required to explain the presence of the different *Salmonella* serotypes and clones isolated from our sampling [2,31,46,47]. Public authorities, managers and health professionals should improve food safety by improving the education, and training in food hygiene of the people responsible for preparing and selling street food.

## Supporting information

S1 Fig Geographic location of the 201 street food stalls sampled in Ouagadougou, Burkina Faso. (PPTX)

S1 Table Characteristics of the 36 *Salmonella* isolates under study. (XLSX)

S2 Table Core-genome multilocus sequence typing analysis of the *S. enterica* serotype Enteritidis strains described in Feasey et al. (reference #^15^). (XLSX)
