# Supplementary figures and images for: Contamination of street food with multidrug-resistant Salmonella, in Ouagadougou, Burkina Faso
Source: PLoS One. 2021 Jun 17;16(6):e0253312. doi: 10.1371/journal.pone.0253312 (PMC8211238; doi:10.1371/journal.pone.0253312)

## Slide 1
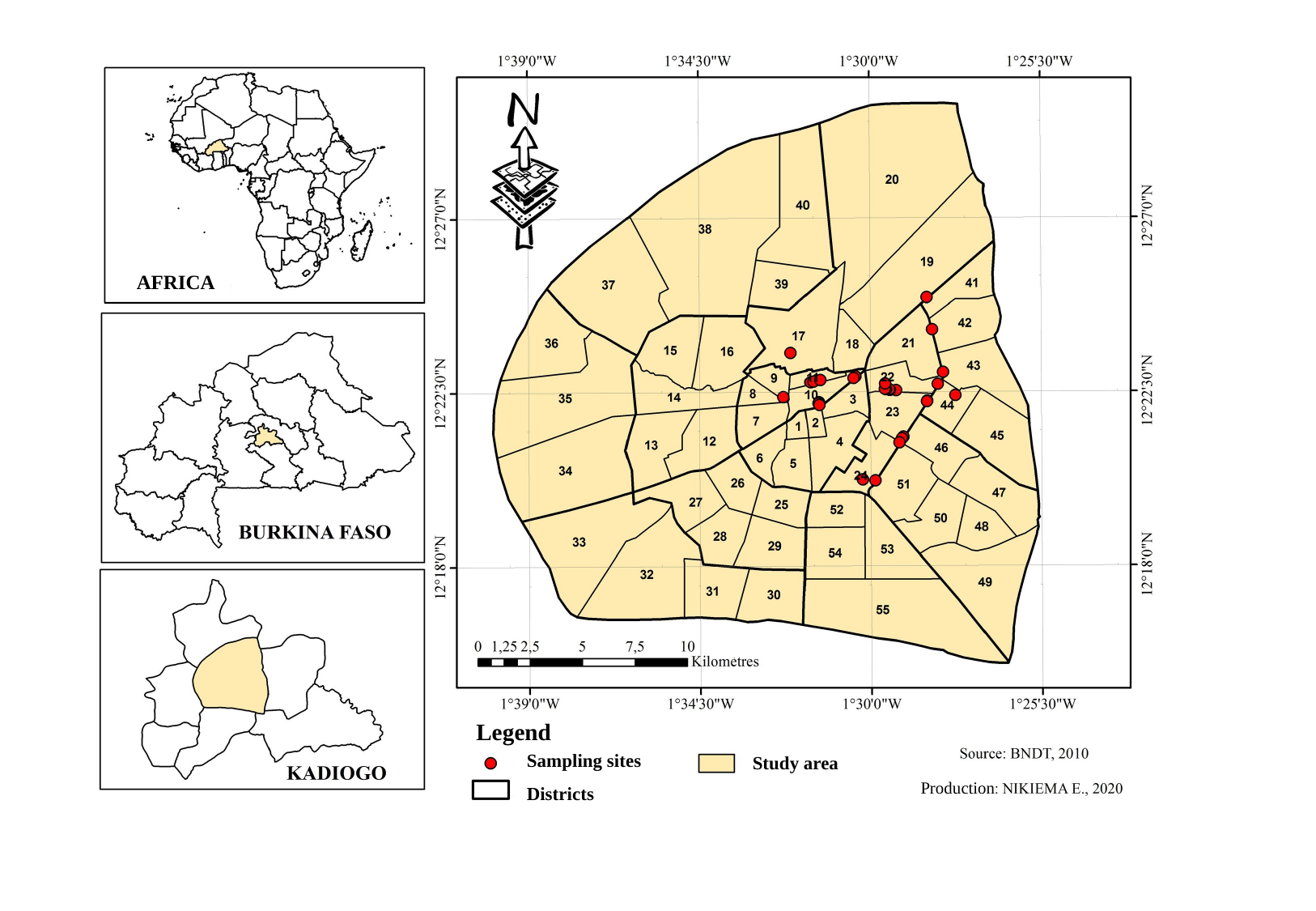

AFRICA
Sampling sites
Districts
Legend
Study area
Production

Supplement: S1 Fig — (PPTX) [file pone.0253312.s001.pptx]
